# Supplementary figures and images for: Divergent genome evolution caused by regional variation in DNA gain and loss between human and mouse
Source: PLoS Comput Biol. 2018 Apr 20;14(4):e1006091. doi: 10.1371/journal.pcbi.1006091 (PMC5931693; doi:10.1371/journal.pcbi.1006091)

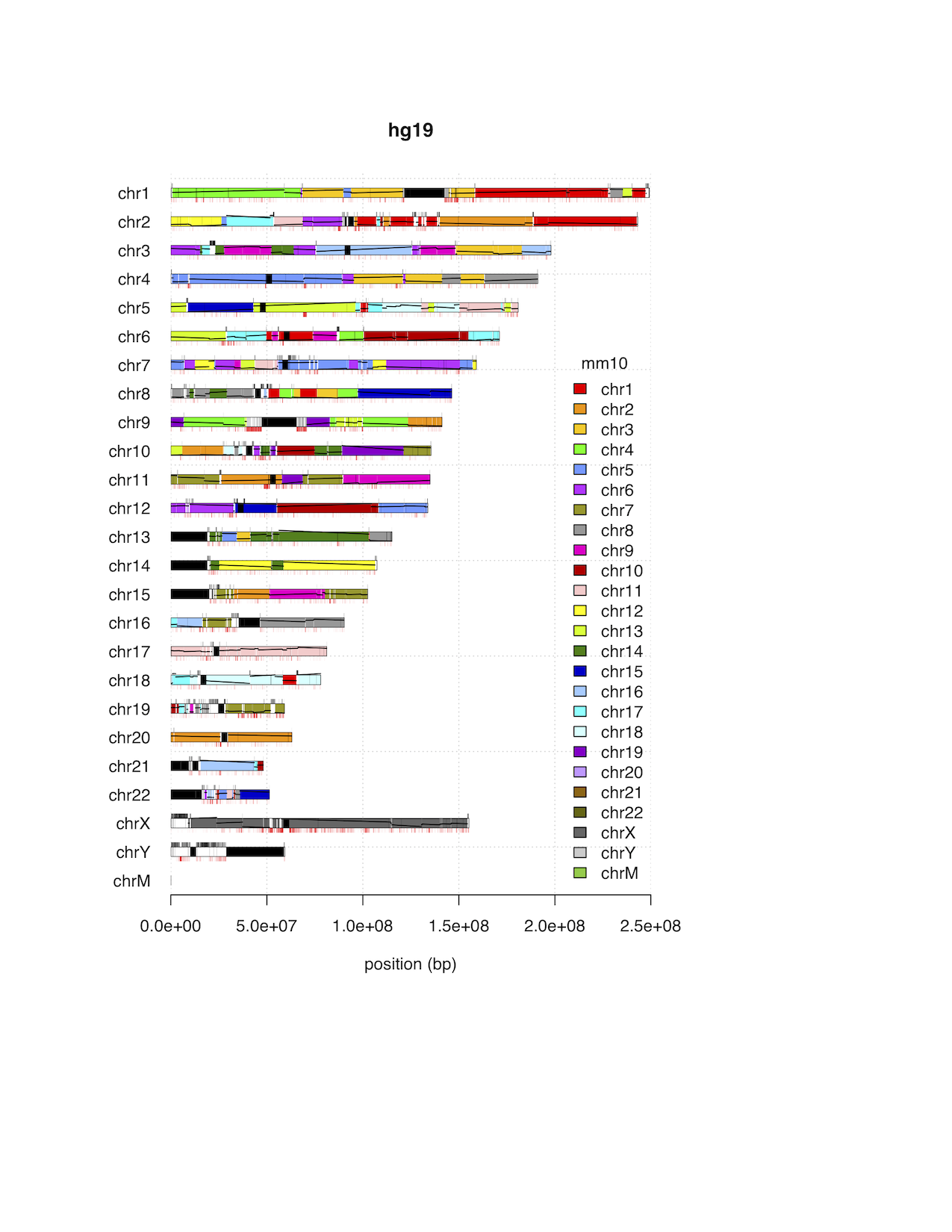

Supplement: S1 Fig — Gaps outside of nets ≥ 10 kb are shown in black above each chromosome. non-RBH regions ≥ 10 kb are shown in red below each chromosome. Assembly gaps are plotted in black within chromosomes. Syntenic blocks are coloured according to which chromosome they belong to in mm10. The trace running through each syntenic block represents its mm10 chromosomal position and orientation, running top to bottom (5′ to 3′). (TIFF) [file pcbi.1006091.s001.tiff]

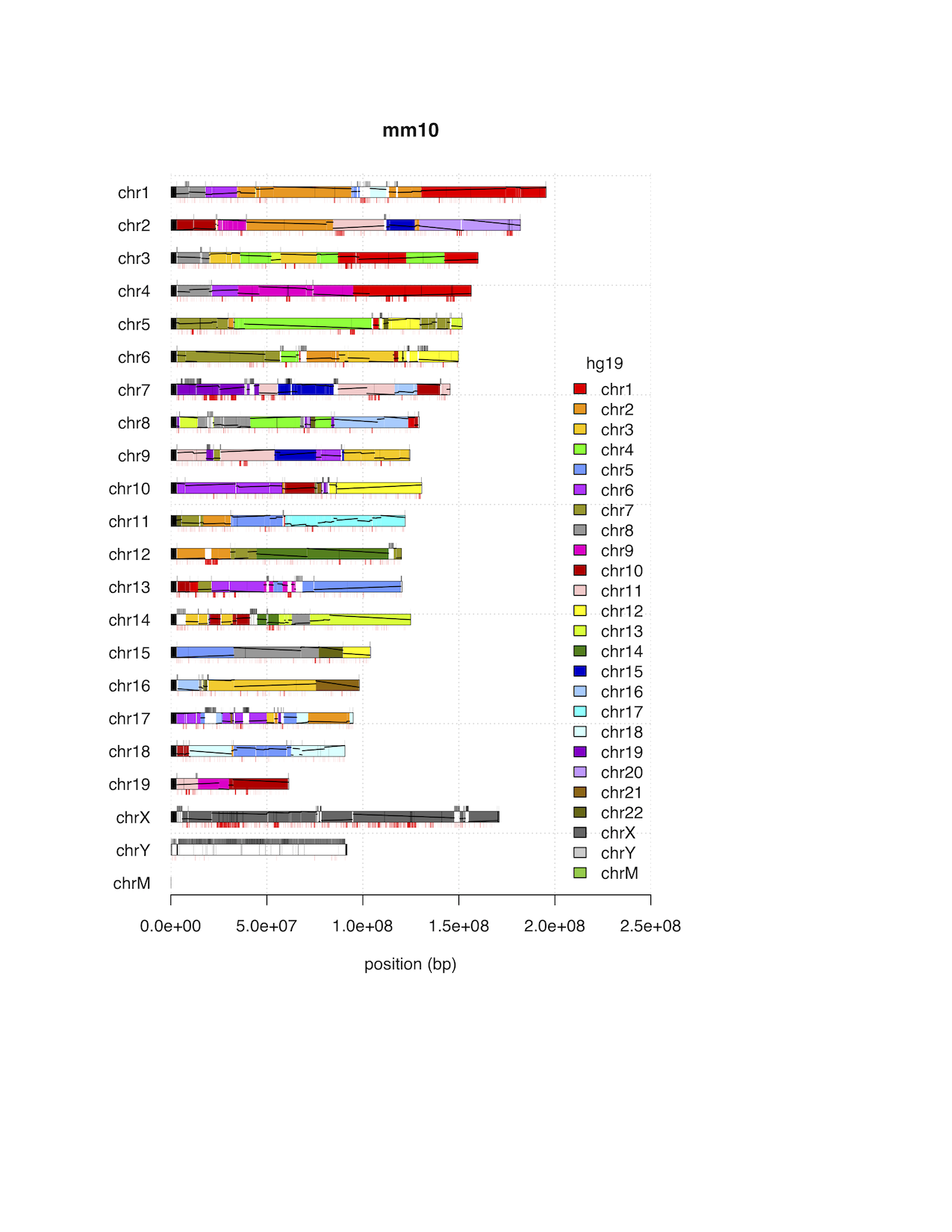

Supplement: S2 Fig — Gaps outside of nets ≥ 10 kb are shown in black above each chromosome. non-RBH regions ≥ 10 kb are shown in red below each chromosome. Assembly gaps are plotted in black within chromosomes. Syntenic blocks are coloured according to which chromosome they belong to in hg19. The trace running through each syntenic block represents its hg19 chromosomal position and orientation, running top to bottom (5′ to 3′). (TIFF) [file pcbi.1006091.s002.tiff]

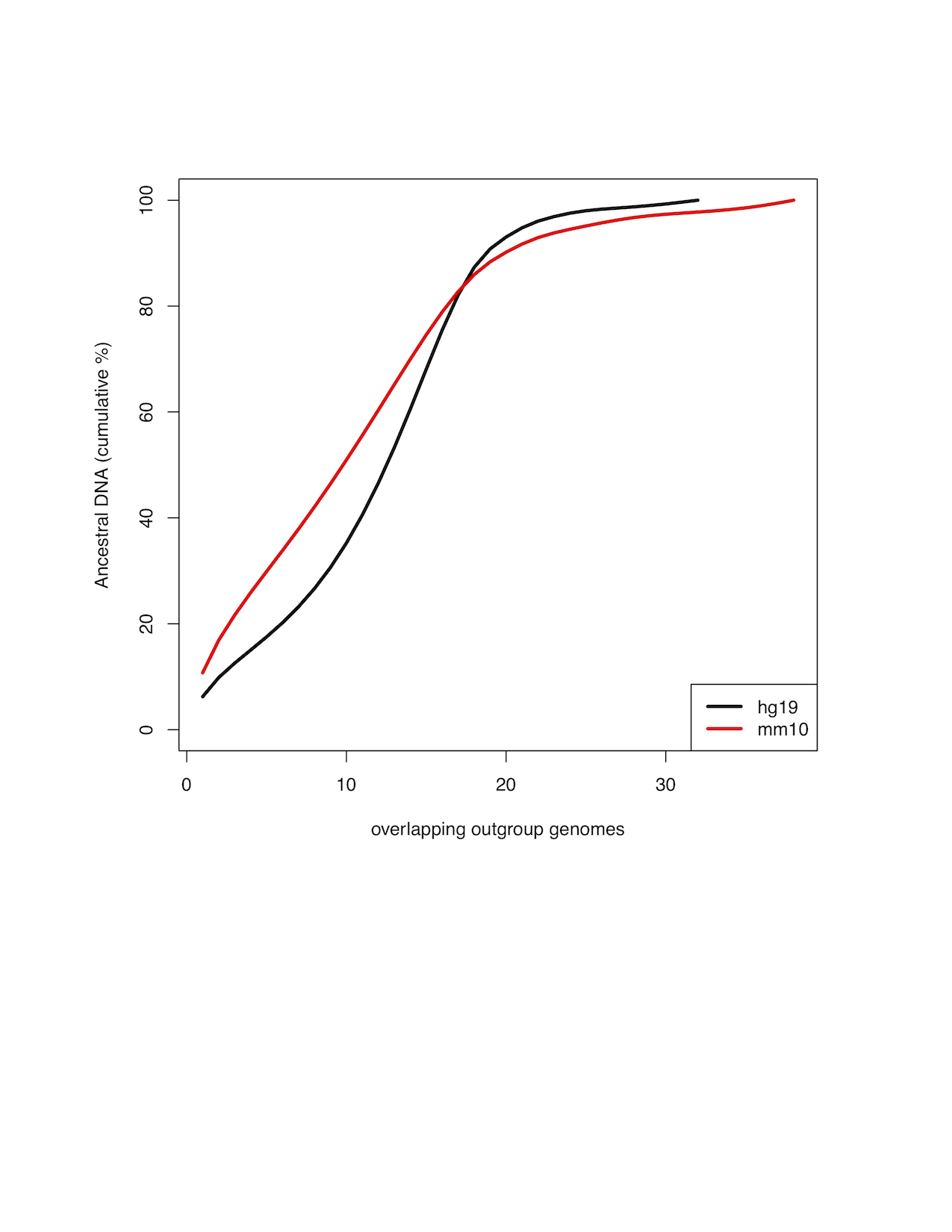

Supplement: S3 Fig — Coverage depth is measured by number of overlapping outgroup species. Ancestral DNA % is the proportion of total bp in hg19 and mm10 that overlap at least one chain-block extracted from an outgroup species. (TIFF) [file pcbi.1006091.s003.tiff]

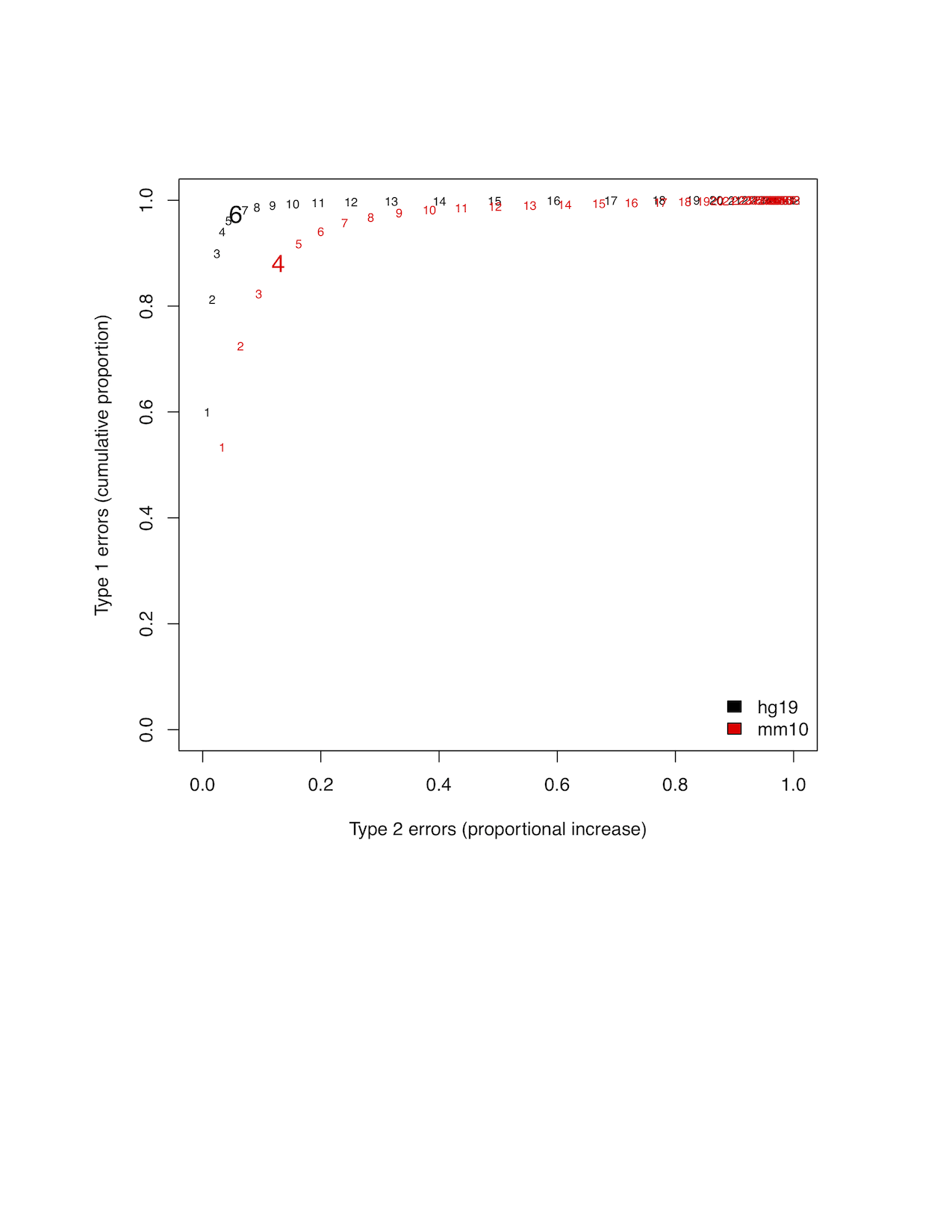

Supplement: S4 Fig — Minimum coverage depth threshold for identifying ancestral elements is plotted against total proportion of identified type 1 errors and the proportional increase in type 2 error rate. Type 1 errors are identified as known recent transposons that overlap chain-blocks extracted from outgroup species. Type 2 errors are identified as chain-blocks between hg19 and mm10 that do not overlap chain-blocks extracted from outgroup species. Type 2 error increase is the reduction in the overlap between outgroup and ingroup (hg19 and mm10) chain-blocks as minimum coverage depth threshold increases. For hg19 and mm10 we chose a minimum coverage depth of 6 and 4 respectively. (TIFF) [file pcbi.1006091.s004.tiff]

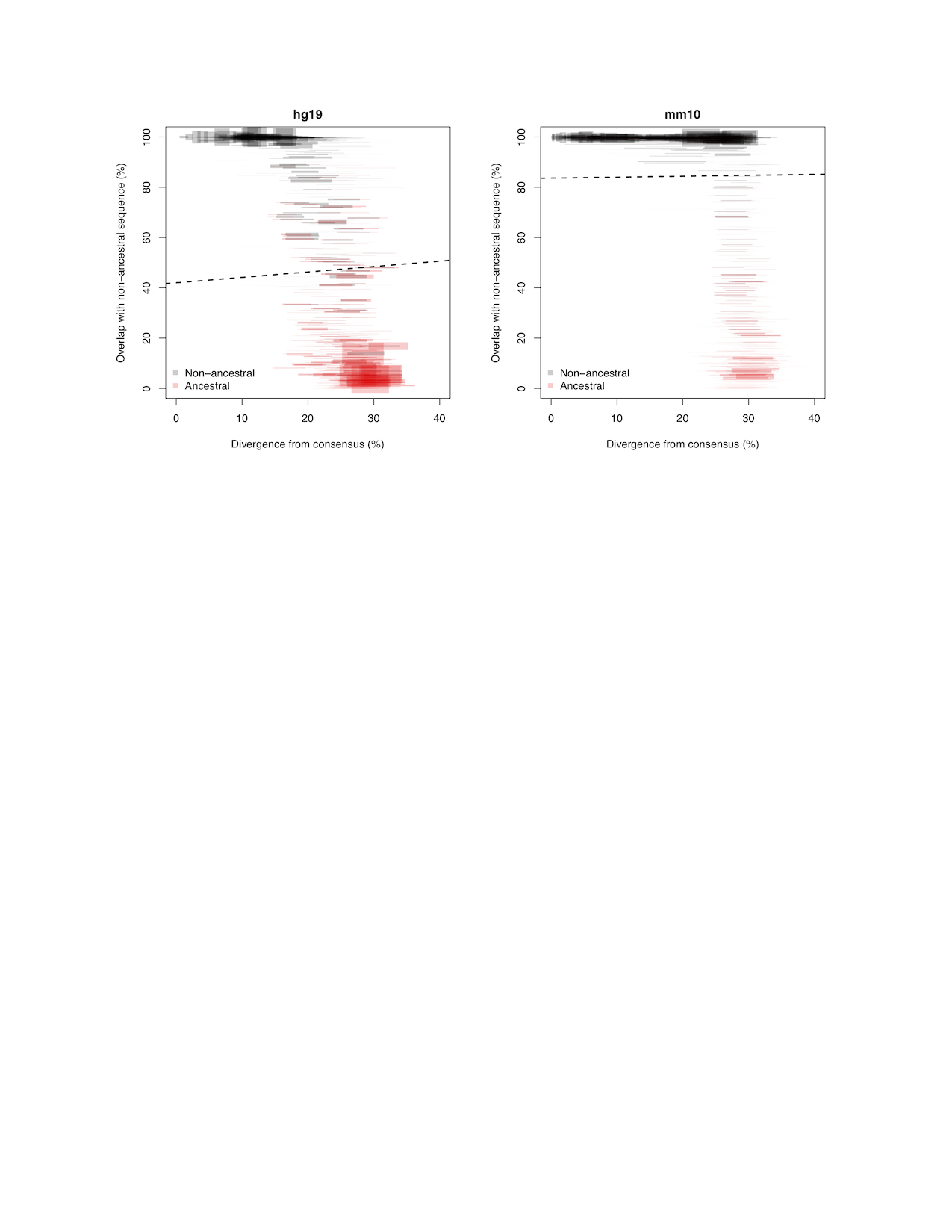

Supplement: S5 Fig — Each rectangle represents the members of a transposon family under our prior recent and ancestral classification. For example, a rectangle coloured black represents the members of a particular transposon family that do not overlap ancestral elements. Rectangle width is the interquartile range of percent divergence from consensus and rectangle height is proportional to total genome coverage. The dotted line is the classification boundary determined by linear discriminant analysis. Rectangles above the line are transposon families classified as recent and rectangles below the line are transposon families classified as ancestral. (TIFF) [file pcbi.1006091.s005.tiff]

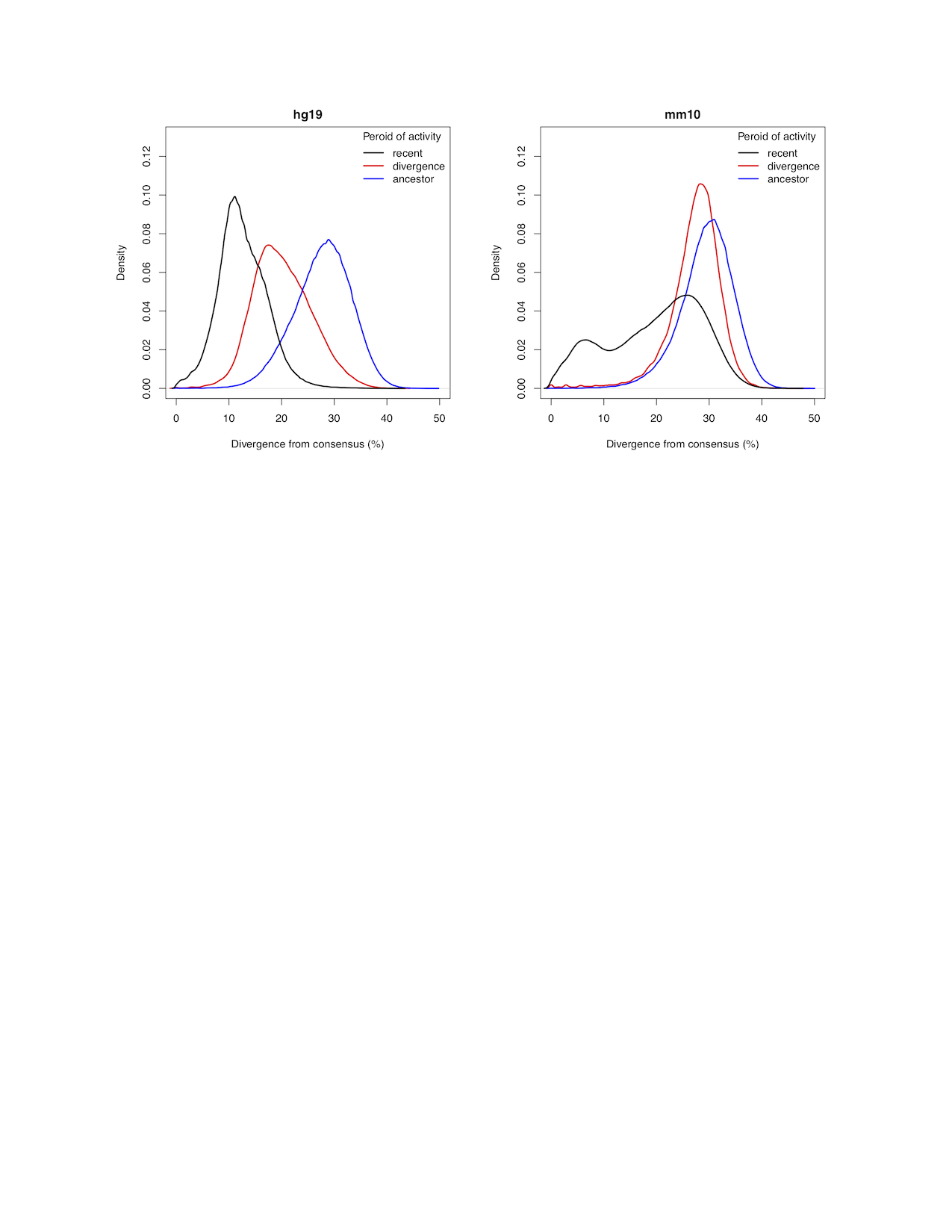

Supplement: S6 Fig — Transposons identified as recently active were classified as recent by our classifier and belong to families not shared between human and mouse. Transposons identified as active during divergence were classified as recent by our classifier and belong to families shared between human and mouse. Transposons identified as active within the ancestor were classified as ancestral by our classifier and belong to families shared between human and mouse. Transposons classified as ancestral by our classifier that belong to families not shared between human and mouse are not shown. (TIFF) [file pcbi.1006091.s006.tiff]

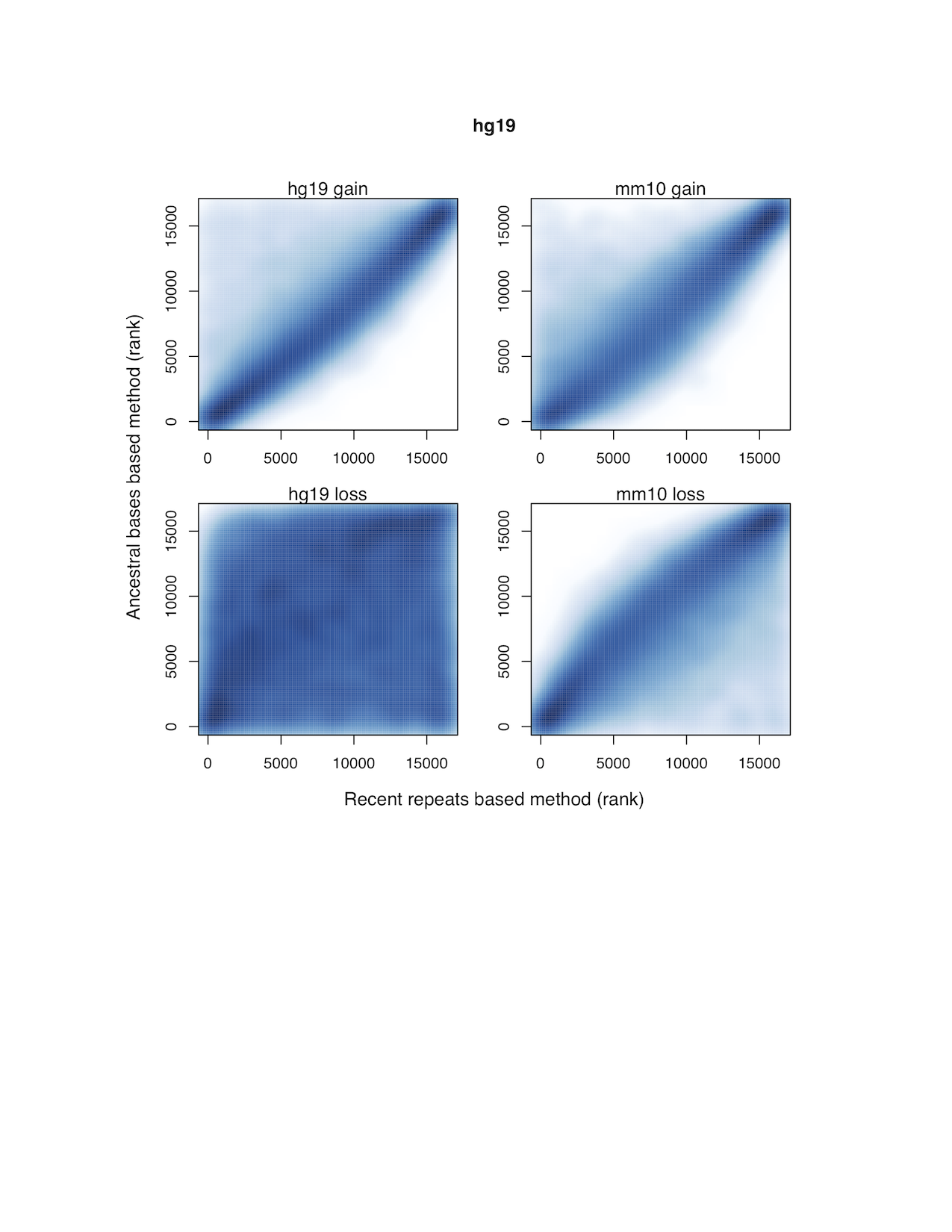

Supplement: S7 Fig — (TIFF) [file pcbi.1006091.s007.tiff]

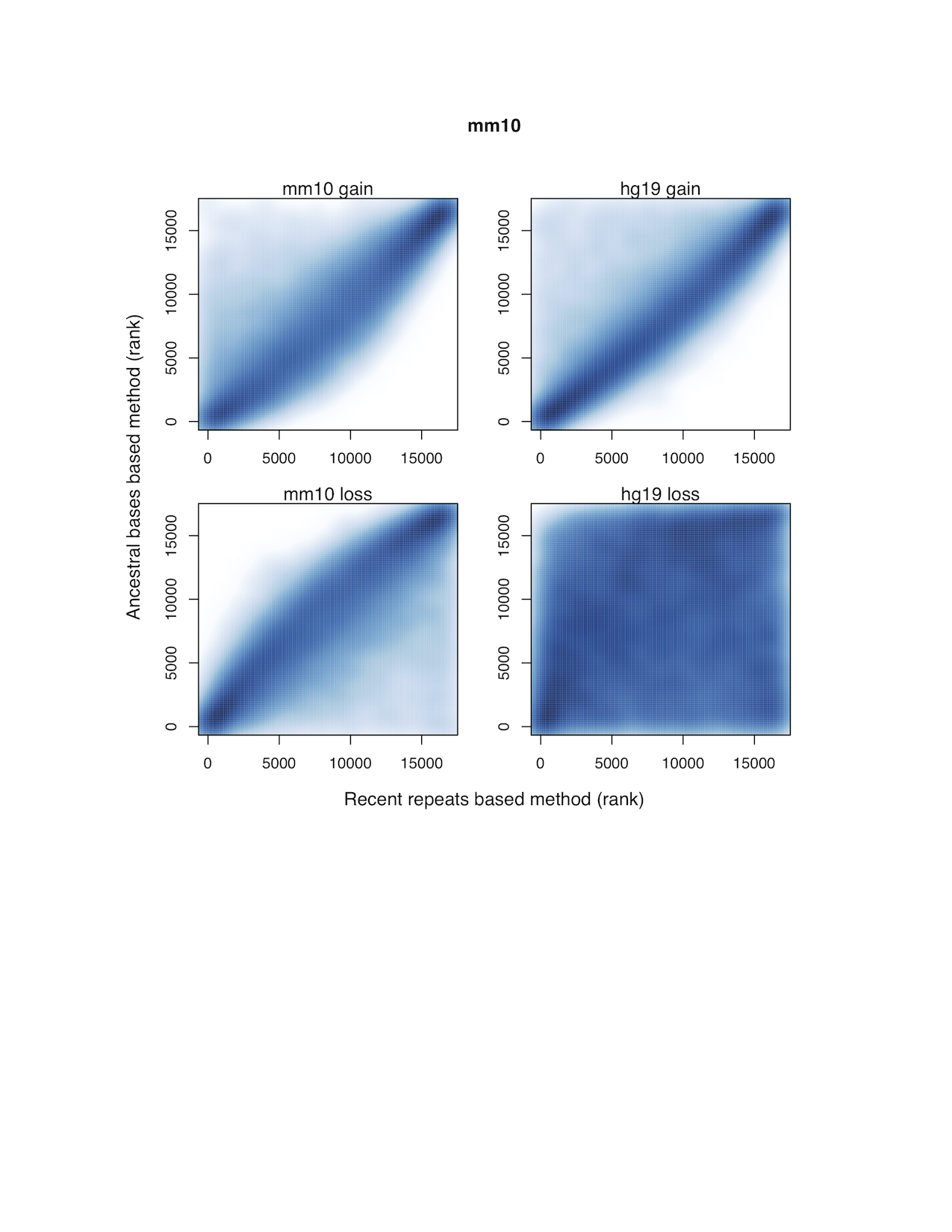

Supplement: S8 Fig — (TIFF) [file pcbi.1006091.s008.tiff]

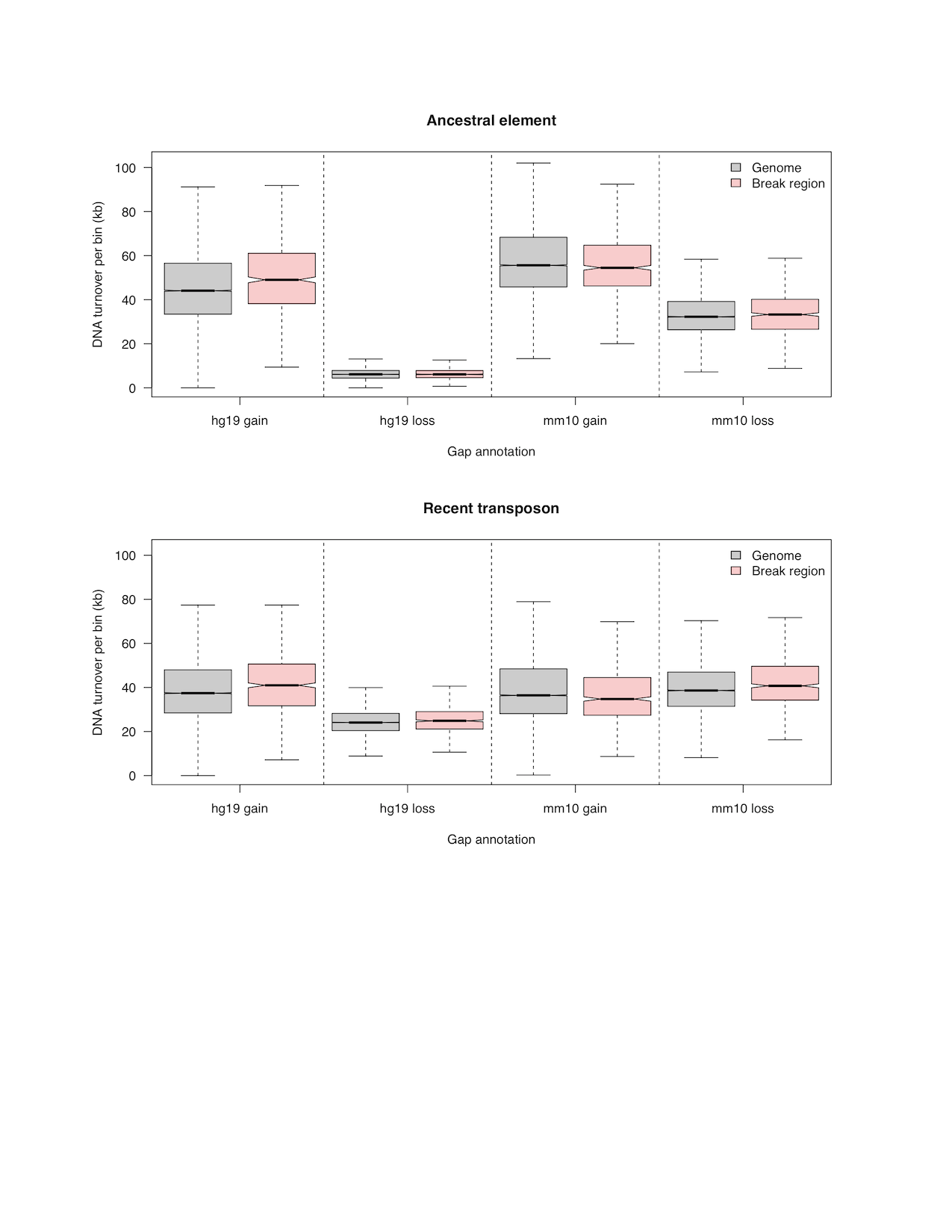

Supplement: S9 Fig — (TIFF) [file pcbi.1006091.s009.tiff]

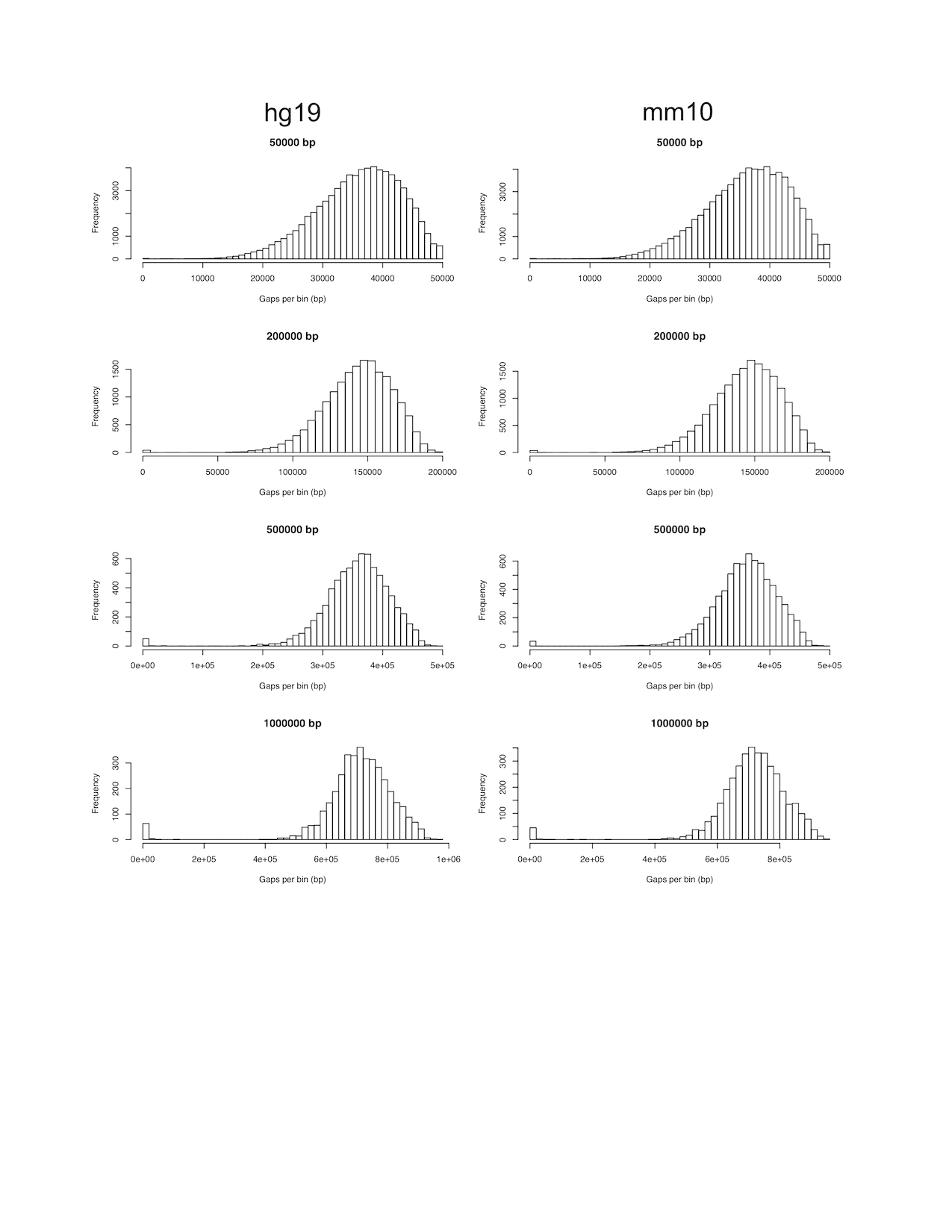

Supplement: S10 Fig — (TIFF) [file pcbi.1006091.s010.tiff]

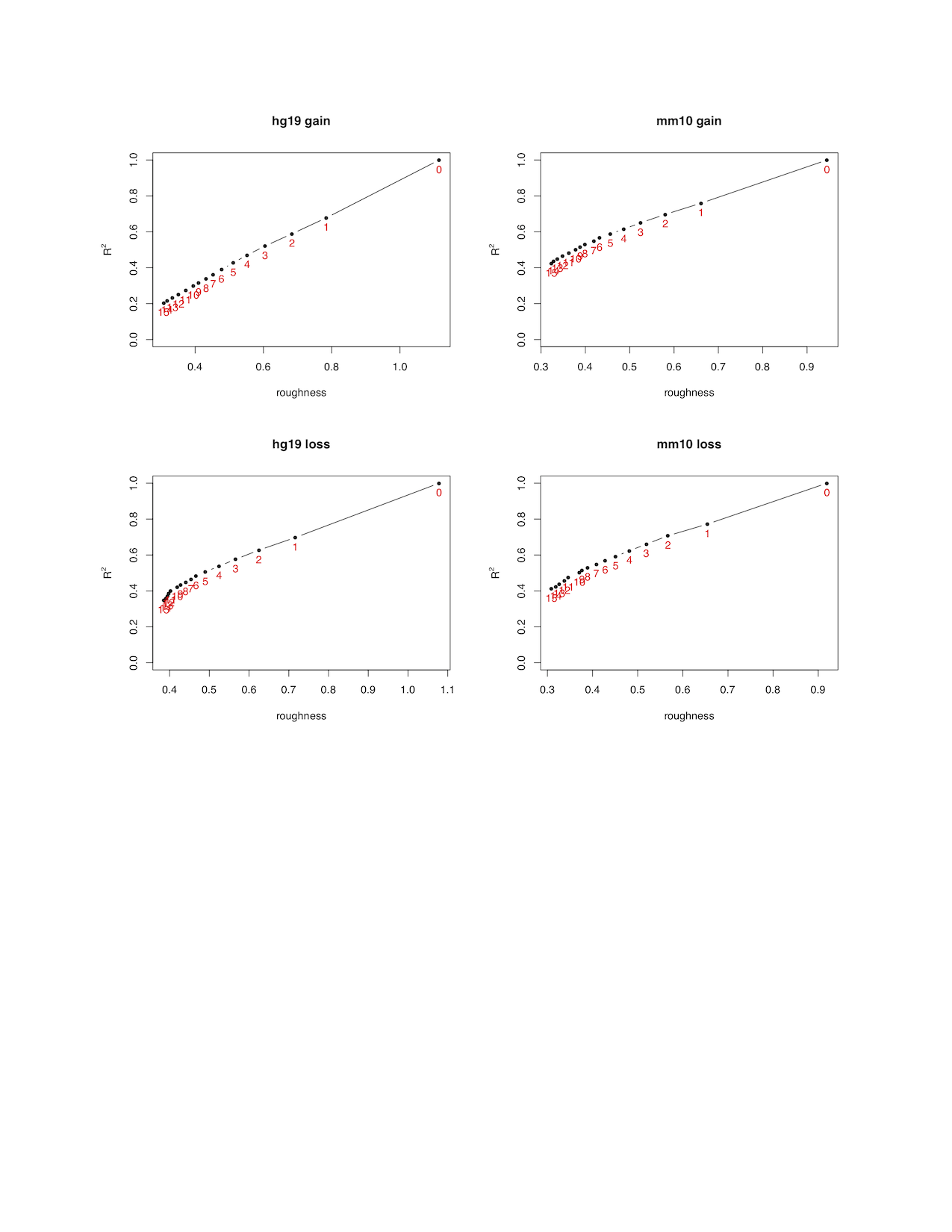

Supplement: S11 Fig — The neighbour distances is shown in red. A neighbour distance of 3 indicates that 3 bins upstream and 3 bins downstream of a particular bin are considered it’s neighbours. R2 is the coefficient of determination between our Gi* values and the actual bin-wise density for a specific gap annotation. “roughness” is calculated as the standard deviation of the differences between adjacent bins, lower values indicate the degree of smoothing caused by increasing the neighbour distance. (TIFF) [file pcbi.1006091.s011.tiff]

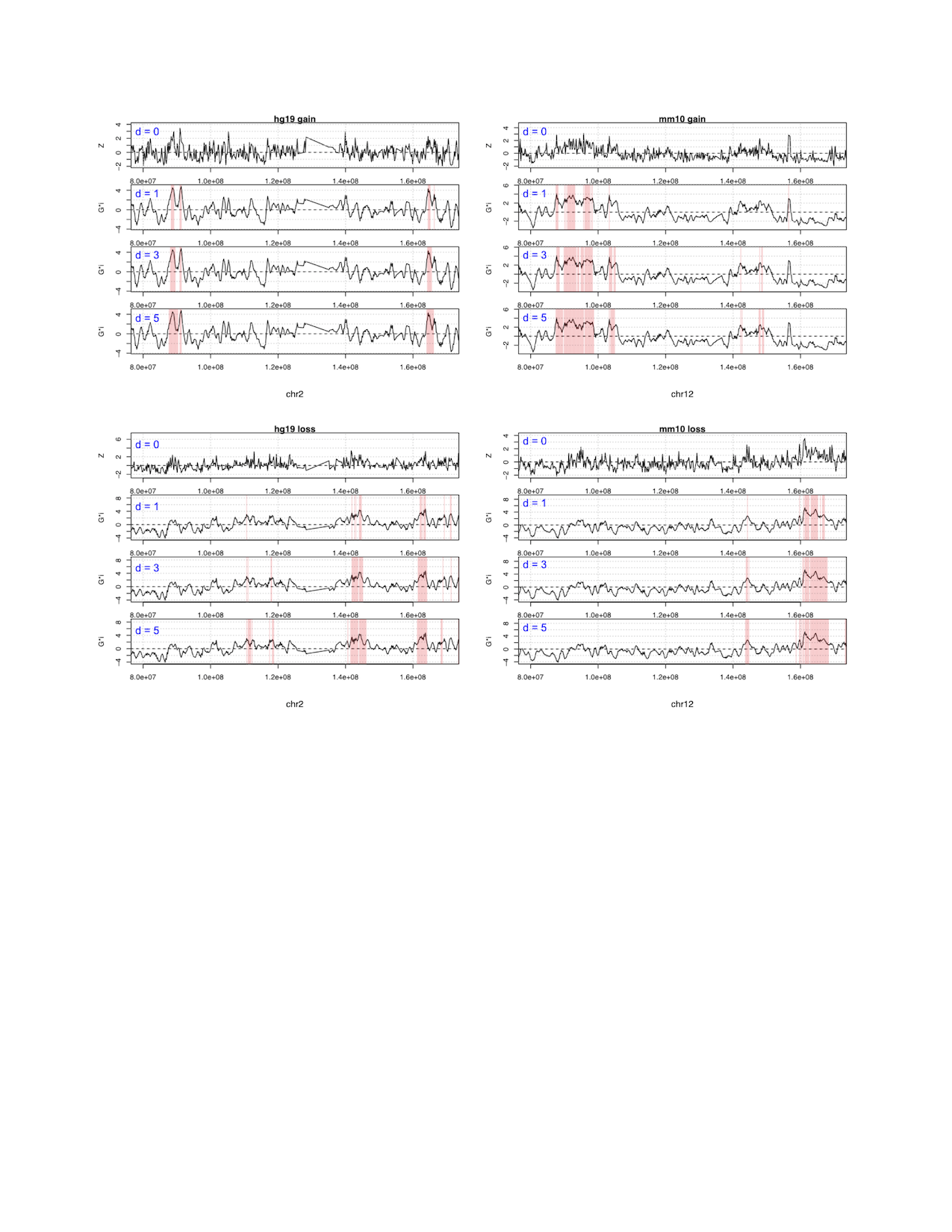

Supplement: S12 Fig — Neighbour distances are indicated in the top left corner of each plot in blue. Hotspots for human and mouse DNA gain and loss are shown in red. (TIFF) [file pcbi.1006091.s012.tiff]

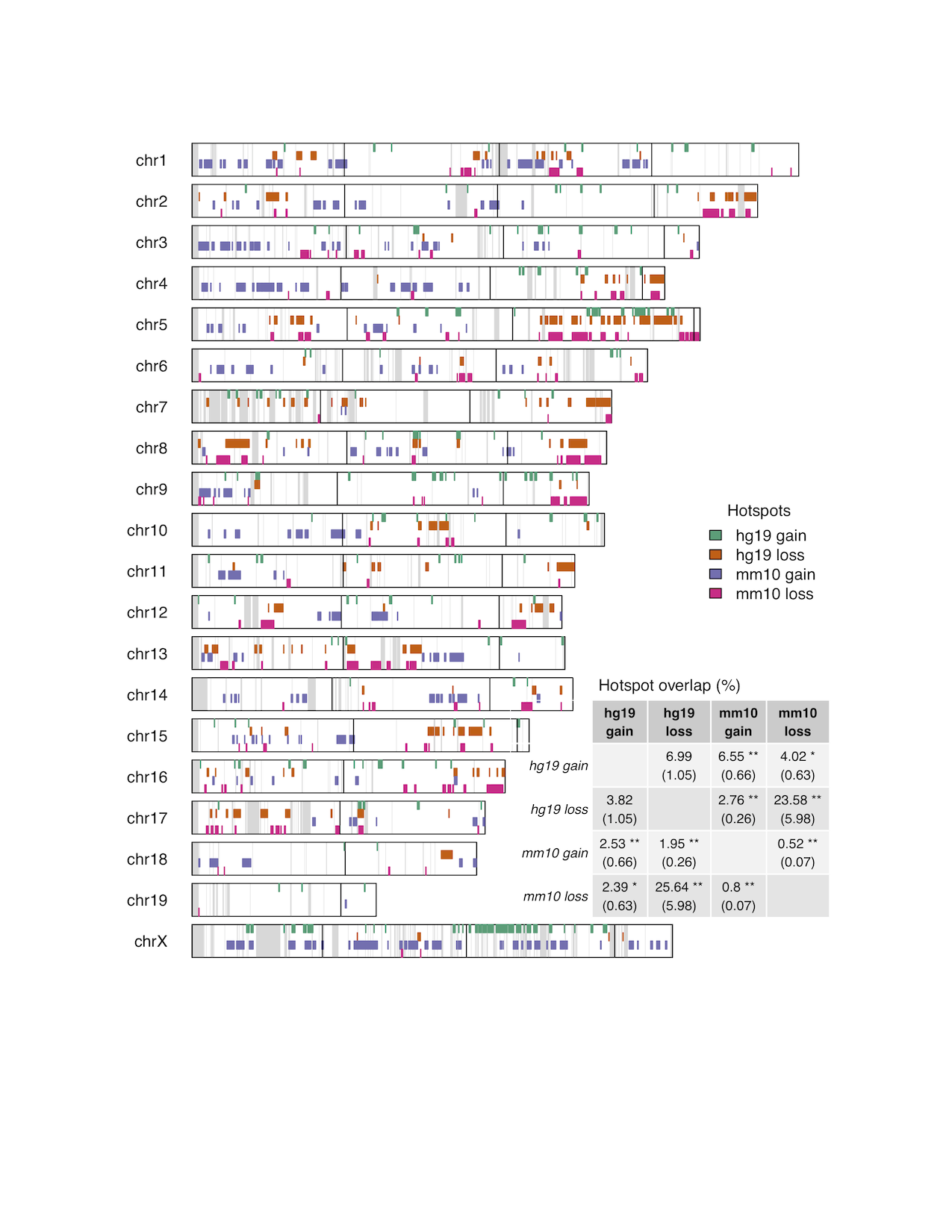

Supplement: S13 Fig — Grey regions indicate bins with ≤ 150 kb of RBH nets and black vertical lines represent 50 Mb on non-synthetic genome. Inset table represents percent overlap of gain and loss hotspots. The percentages were calculated using the hotspots labelled in each row as the denominator. ‘*’ and ‘**’ represent p-values below .05 and .01 respectively based on the Fisher statistic. (TIFF) [file pcbi.1006091.s013.tiff]

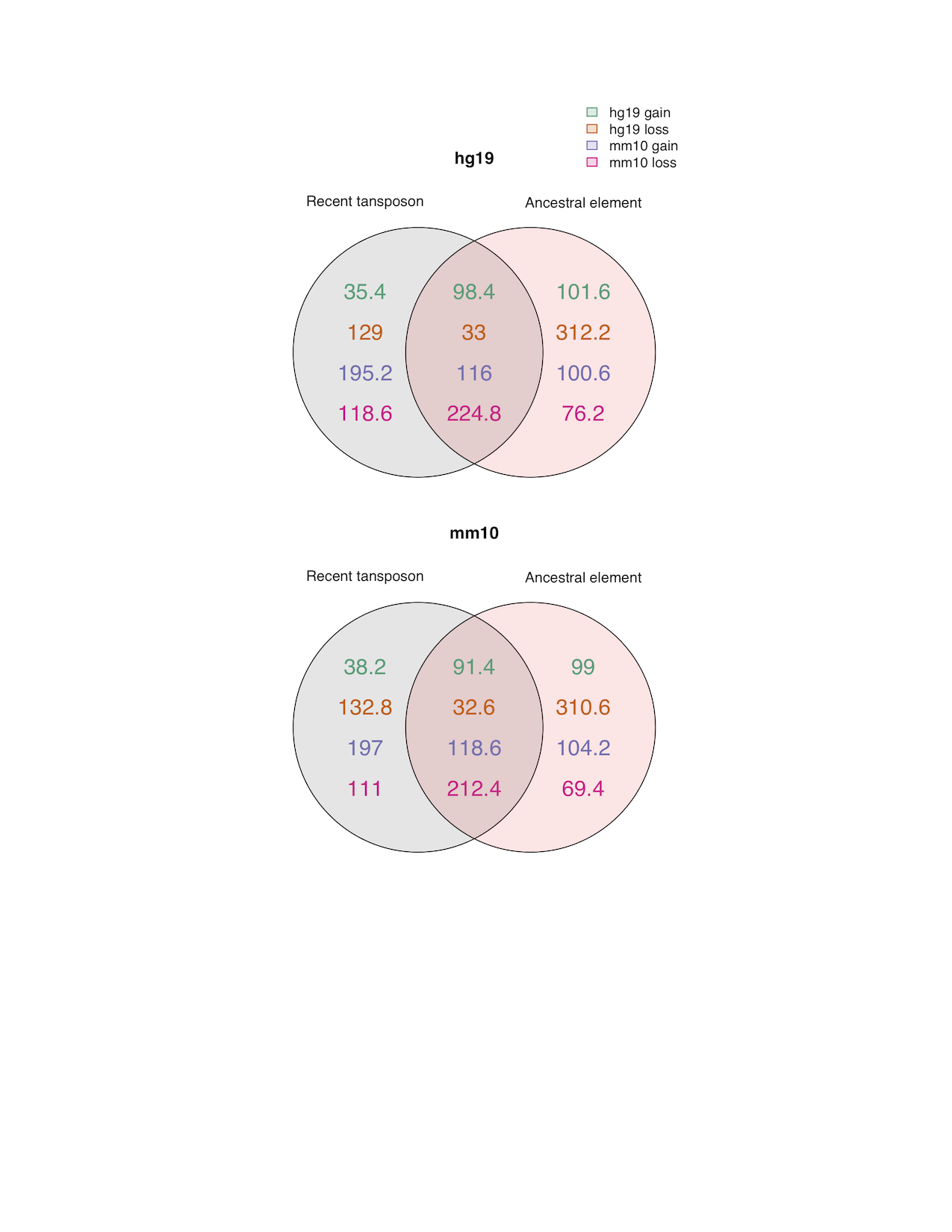

Supplement: S14 Fig — Regions identified as coldspots (Mb) for DNA gain or loss using the Gi* statistic in each genome. (TIFF) [file pcbi.1006091.s014.tiff]

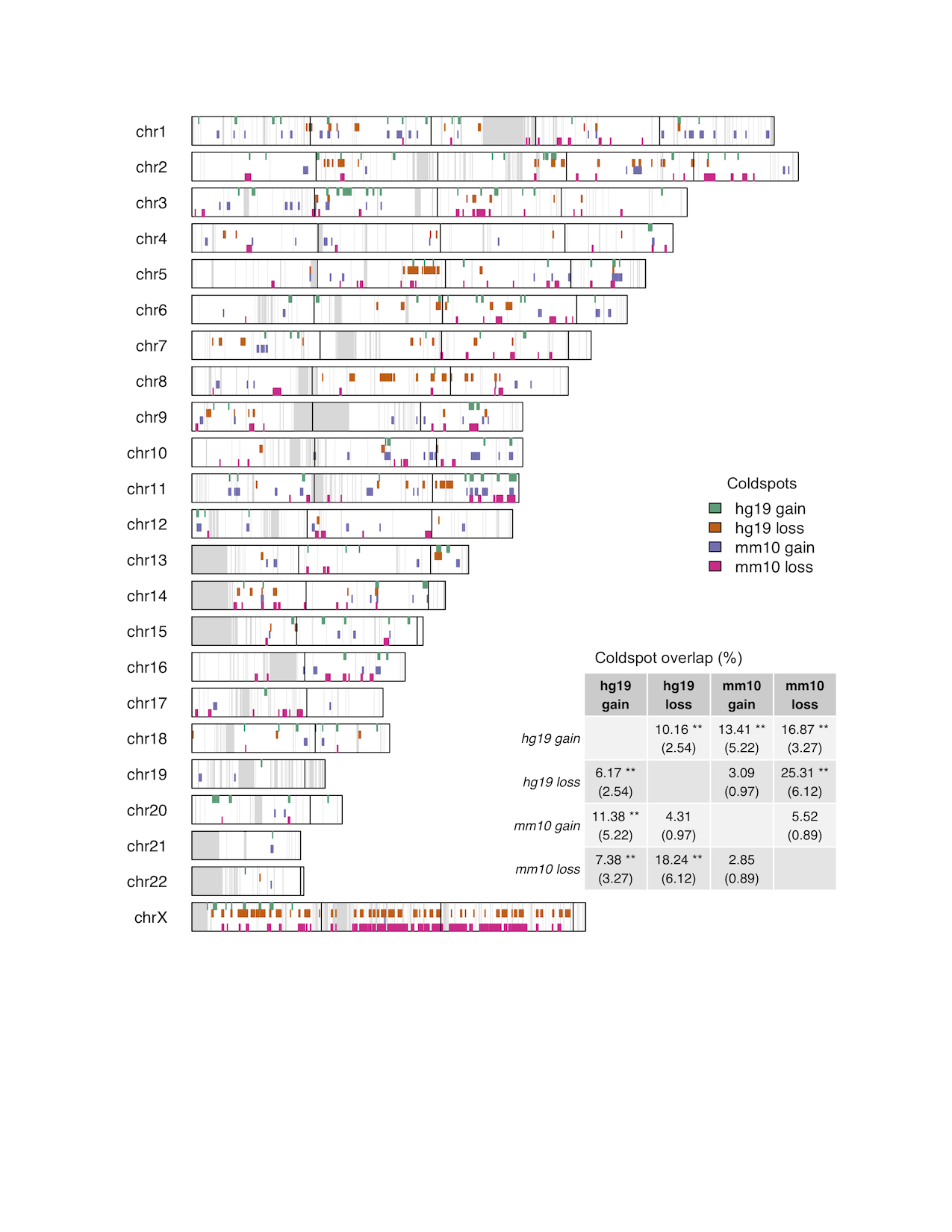

Supplement: S15 Fig — Grey regions indicate bins with ≤ 150 kb of RBH nets and black vertical lines represent 50 Mb on non-synthetic genome. Inset table represents percent overlap of gain and loss coldspots. The percentages were calculated using the coldspots labelled in each row as the denominator. ‘*’ and ‘**’ represent p-values below .05 and .01 respectively based on the Fisher statistic. (TIFF) [file pcbi.1006091.s015.tiff]

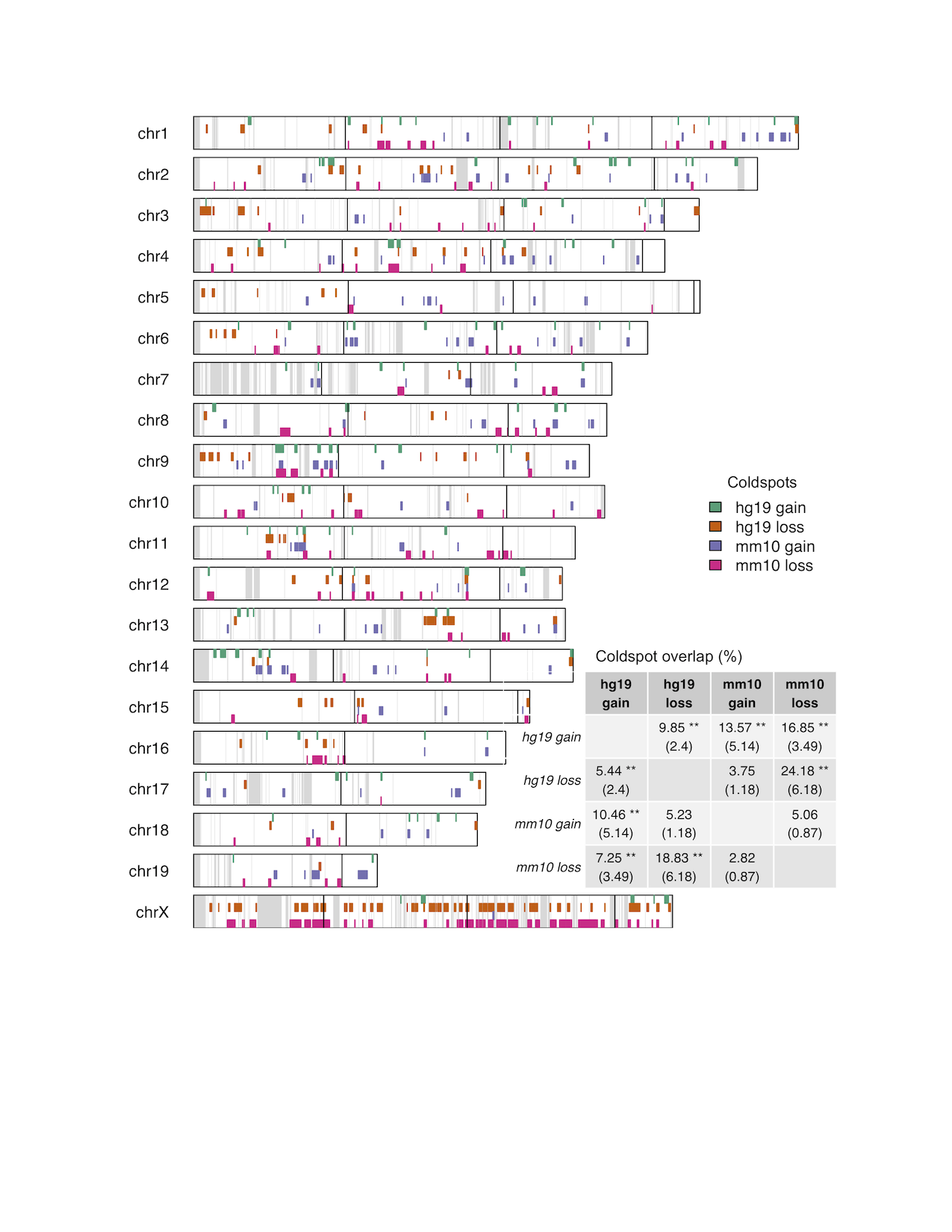

Supplement: S16 Fig — Grey regions indicate bins with ≤ 150 kb of RBH nets and black vertical lines represent 50 Mb on non-synthetic genome. Inset table represents percent overlap of gain and loss coldspots. The percentages were calculated using the coldspots labelled in each row as the denominator. ‘*’ and ‘**’ represent p-values below .05 and .01 respectively based on the Fisher statistic. (TIFF) [file pcbi.1006091.s016.tiff]

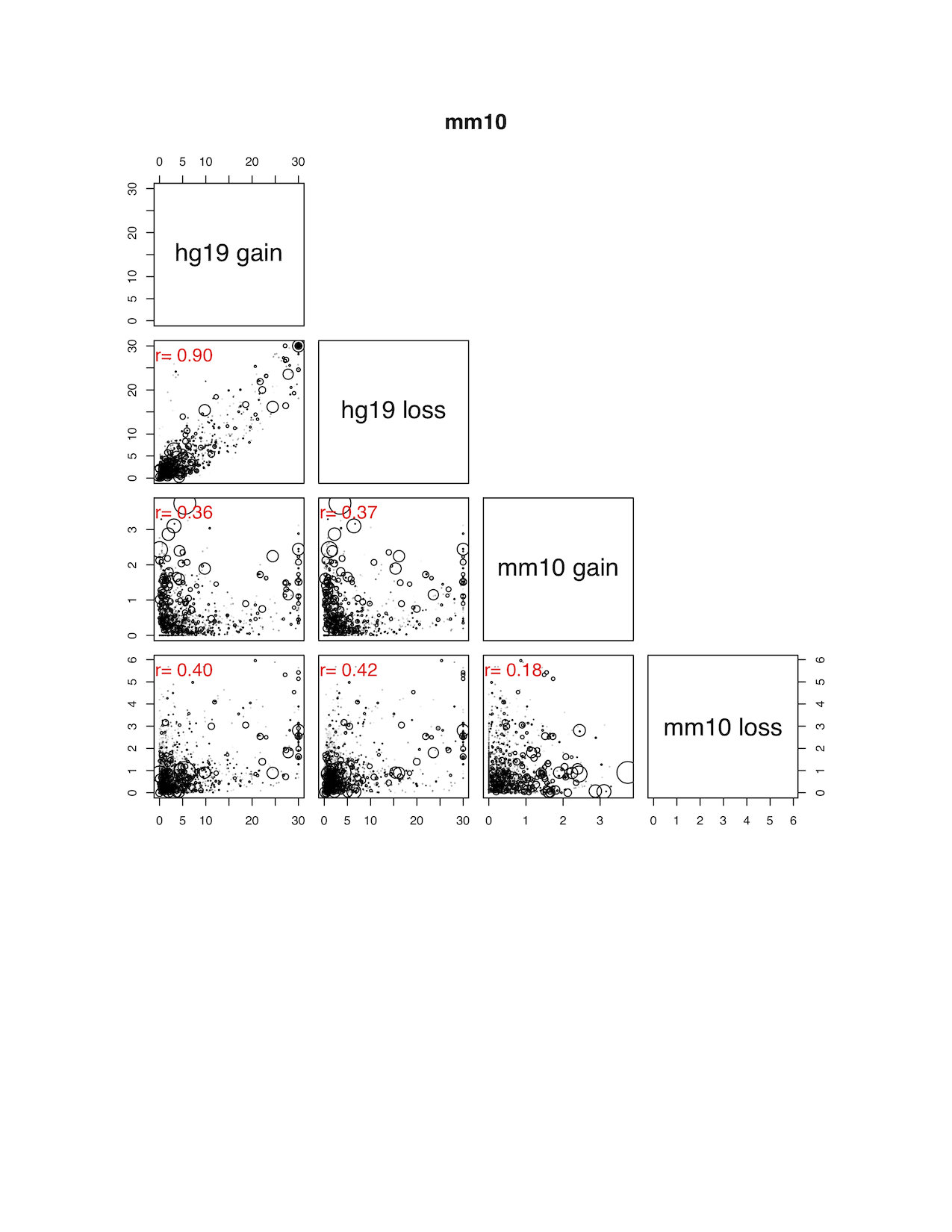

Supplement: S17 Fig — The axes are marked according to -log10 P-values. The size of points represents the total umber of annotations for each GO term. In red is the Pearson correlation coefficient. (TIFF) [file pcbi.1006091.s017.tiff]

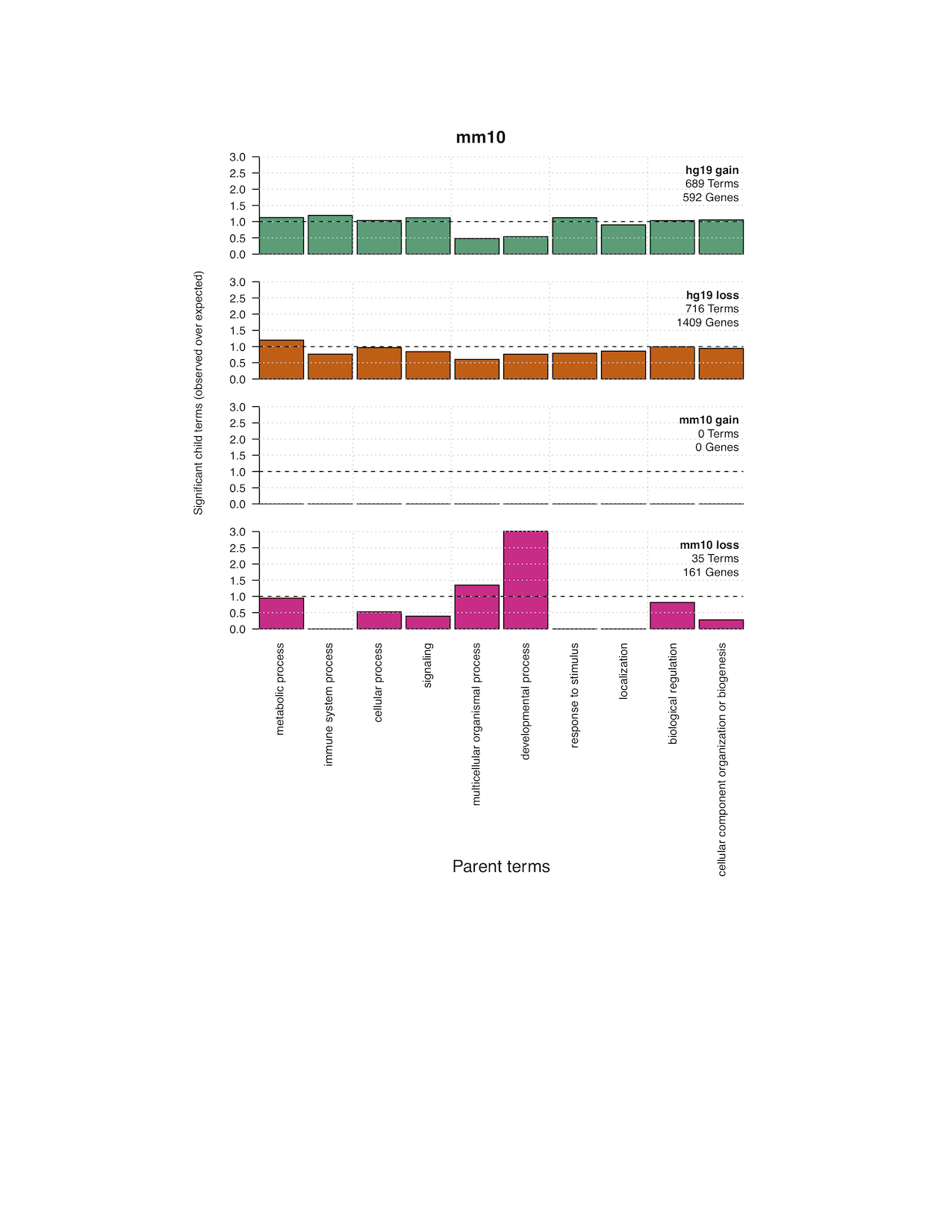

Supplement: S18 Fig — Parent terms were the top level biological process GO terms while child terms were those beneath each parent term. Only Parent terms whose children make up > 5% of all terms in the genome are shown. Child terms were identified as significant at a FDR < 0.05 based on a Fisher test using the ‘classic’ algorithm. The Y axis represents the proportion of significant child terms belonging to a particular parent (observed), divided by the proportion of all child terms in the genome that belong to that same parent term (expected). Also shown is the number of non-redundant GO terms and genes annotated with significant GO terms for each gap annotation. (TIFF) [file pcbi.1006091.s018.tiff]

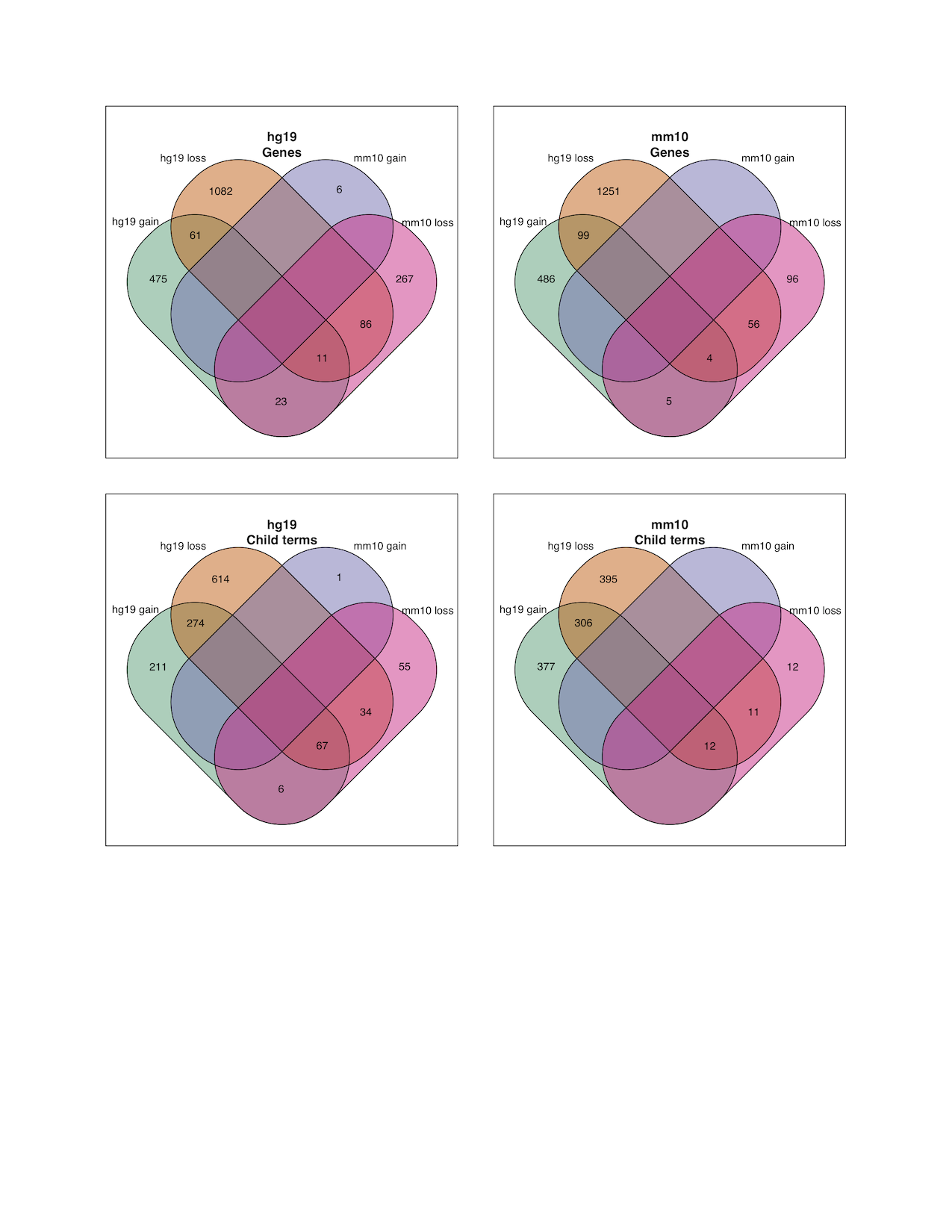

Supplement: S19 Fig — GO terms were identified as significant at a FDR < 0.05 based on a Fisher test using the ‘classic’ algorithm. Annotated genes are genes that have been annotated with at least one of the significant GO terms. GO term lists and gene lists in each set are non-redundant. (TIFF) [file pcbi.1006091.s019.tiff]

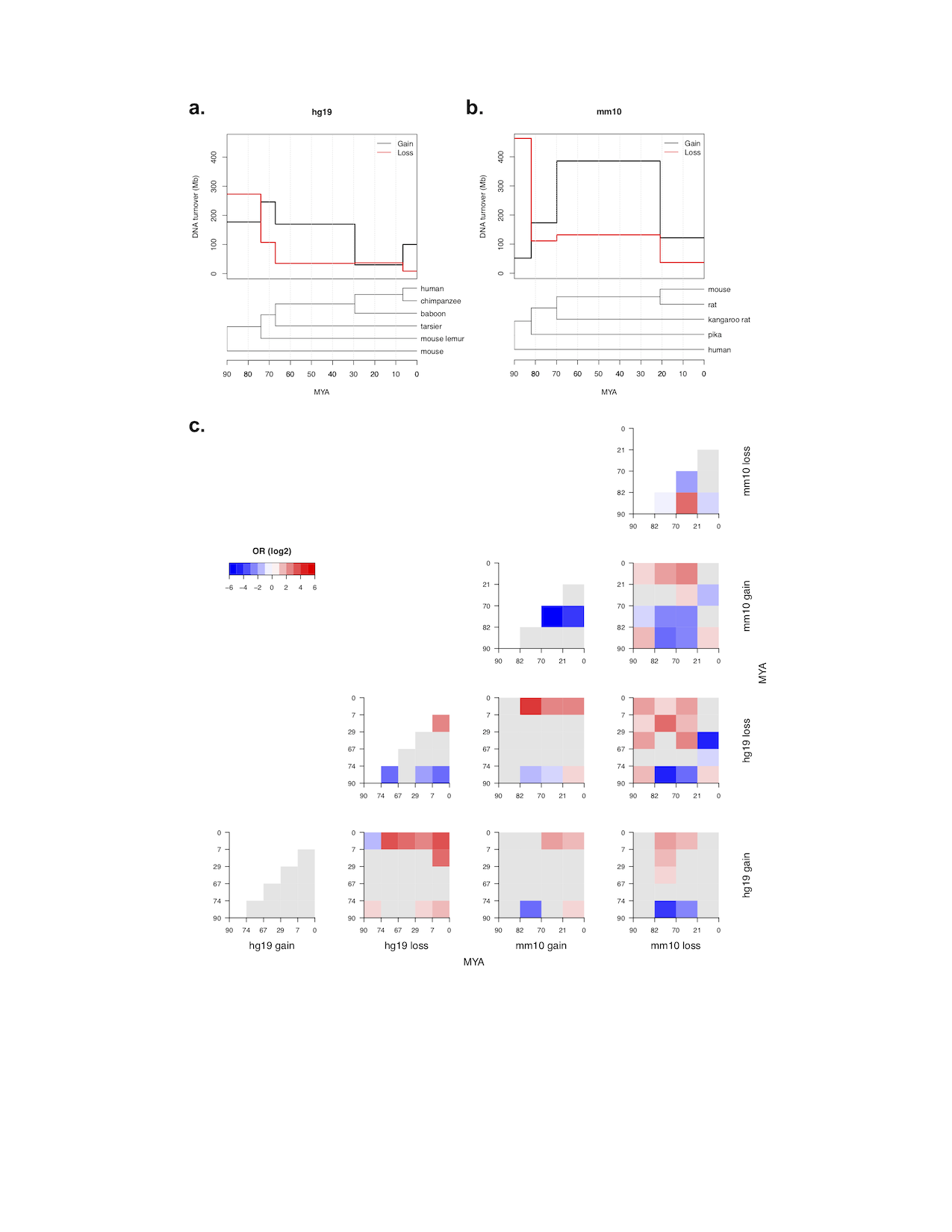

Supplement: S20 Fig — Amount of DNA gain and loss in Mb that occurred since the human (a) and mouse (b) divergence event. Levels of DNA turnover reflect the amount of DNA gain or loss that can be attributed to each branch pictured below in the phylogenetic trees. Divergence times for the phylogenetic trees were calculated using time tree’s “estimated divergence time”. Overlap between DNA gain and loss hotspots in the mm10 genomic background specific to each divergence event (c). Significant positive and negative associations based on Fisher’s exact test (FDR < 0.05) are coloured according to their log2 odds ratio (OR). Numbers on each plot’s x and y axis represent time periods which individual DNA gains and losses were assigned to. (TIFF) [file pcbi.1006091.s020.tiff]

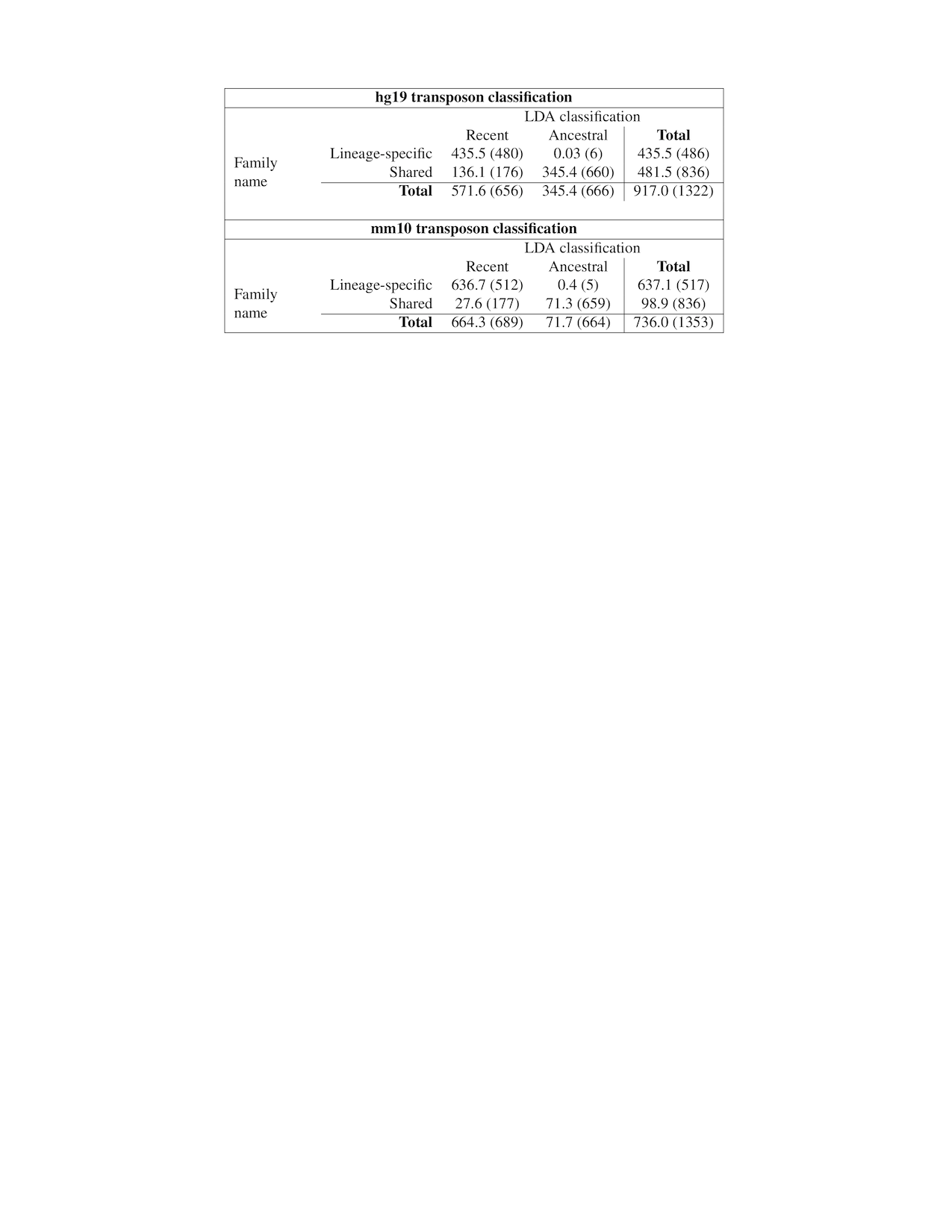

Supplement: S1 Table — Transposon classification compares our LDA classifier against shared and lineage-specific transposon family names. Presented is the total Mb transposon coverage with number of families in brackets. (TIFF) [file pcbi.1006091.s021.tiff]

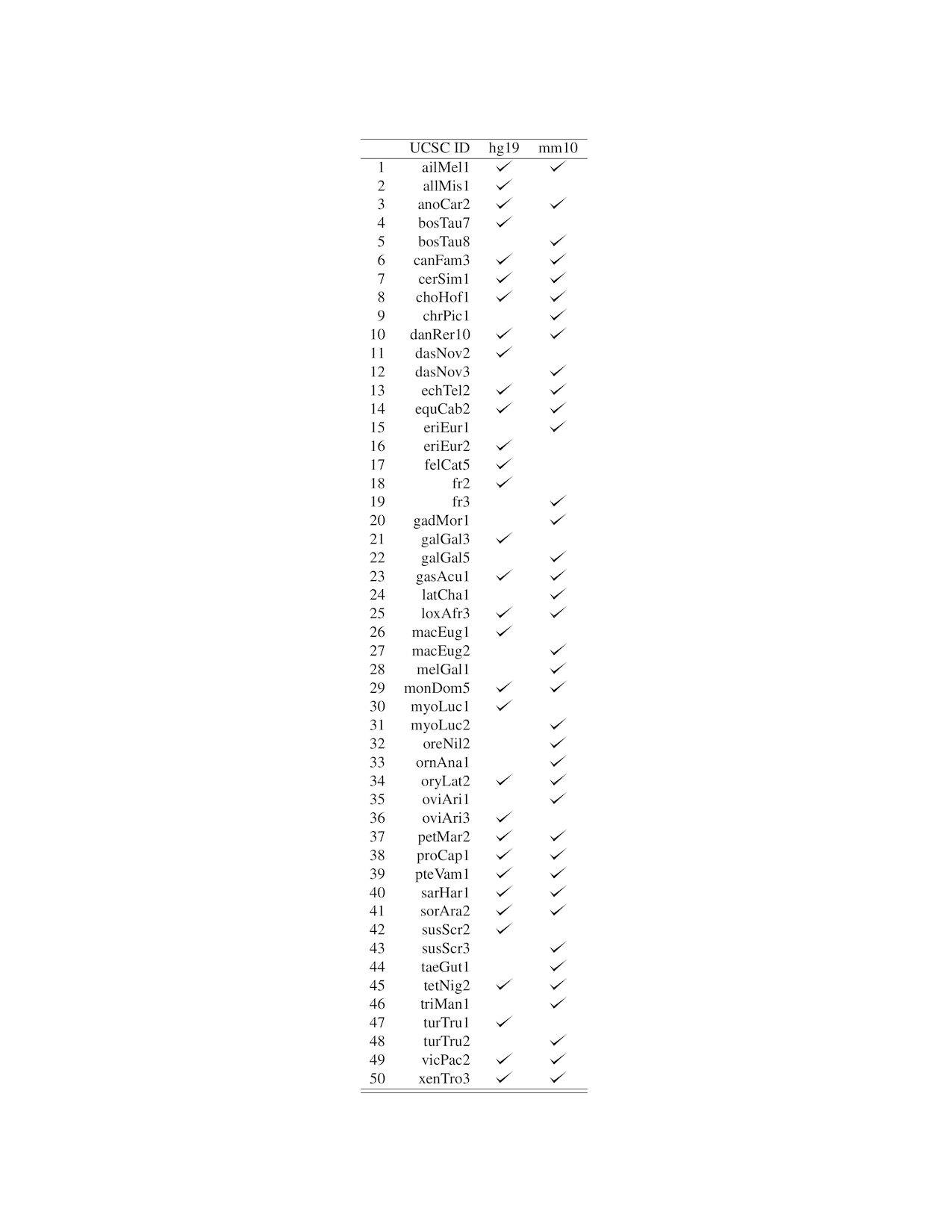

Supplement: S2 Table — (TIFF) [file pcbi.1006091.s022.tiff]

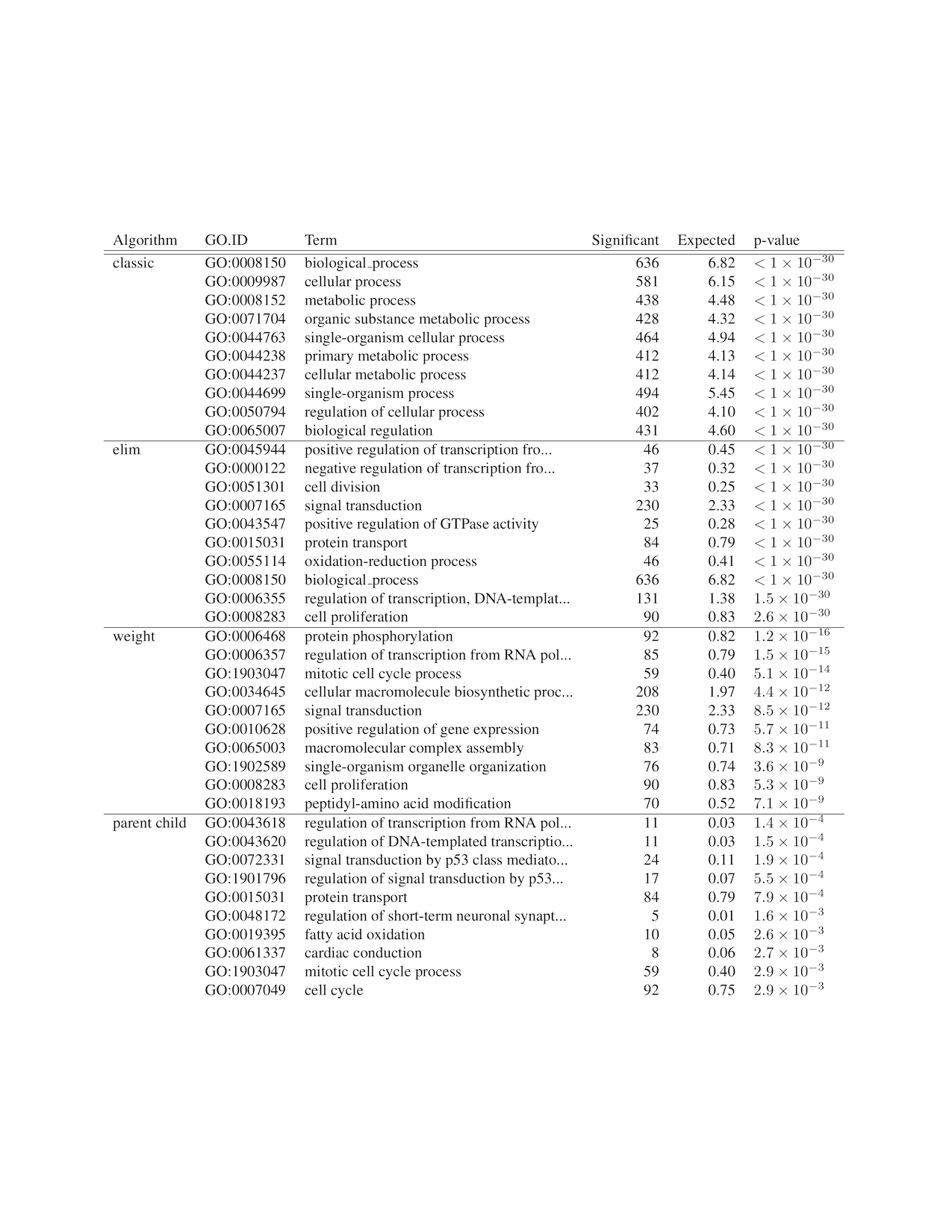

Supplement: S3 Table — P-values for each GO term were calculated using the fisher statistic combined with one of four separate algorithms that each take the GO hierarchy into account (described in Methods). (TIFF) [file pcbi.1006091.s023.tiff]

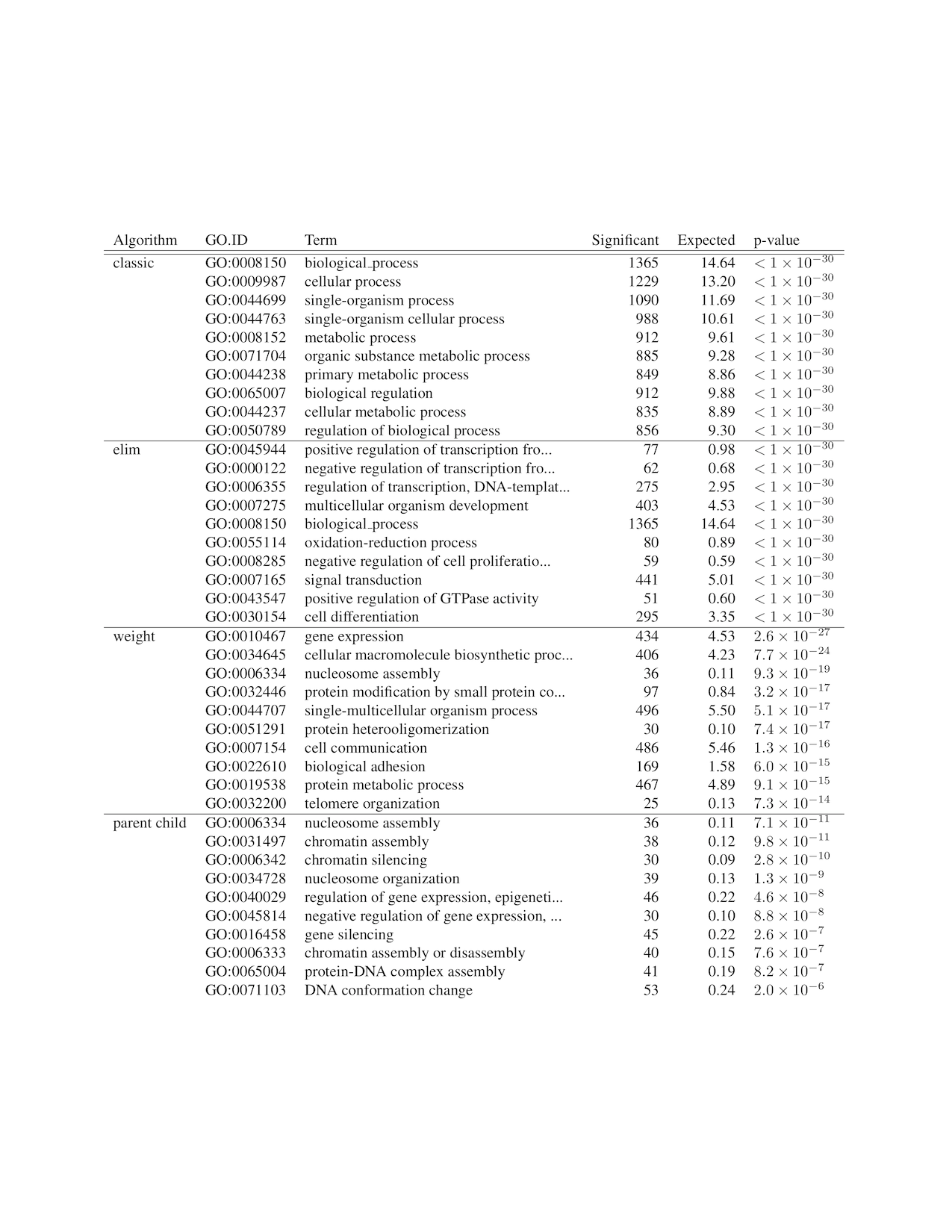

Supplement: S4 Table — P-values for each GO term were calculated using the fisher statistic combined with one of four separate algorithms that each take the GO hierarchy into account (described in Methods). (TIFF) [file pcbi.1006091.s024.tiff]

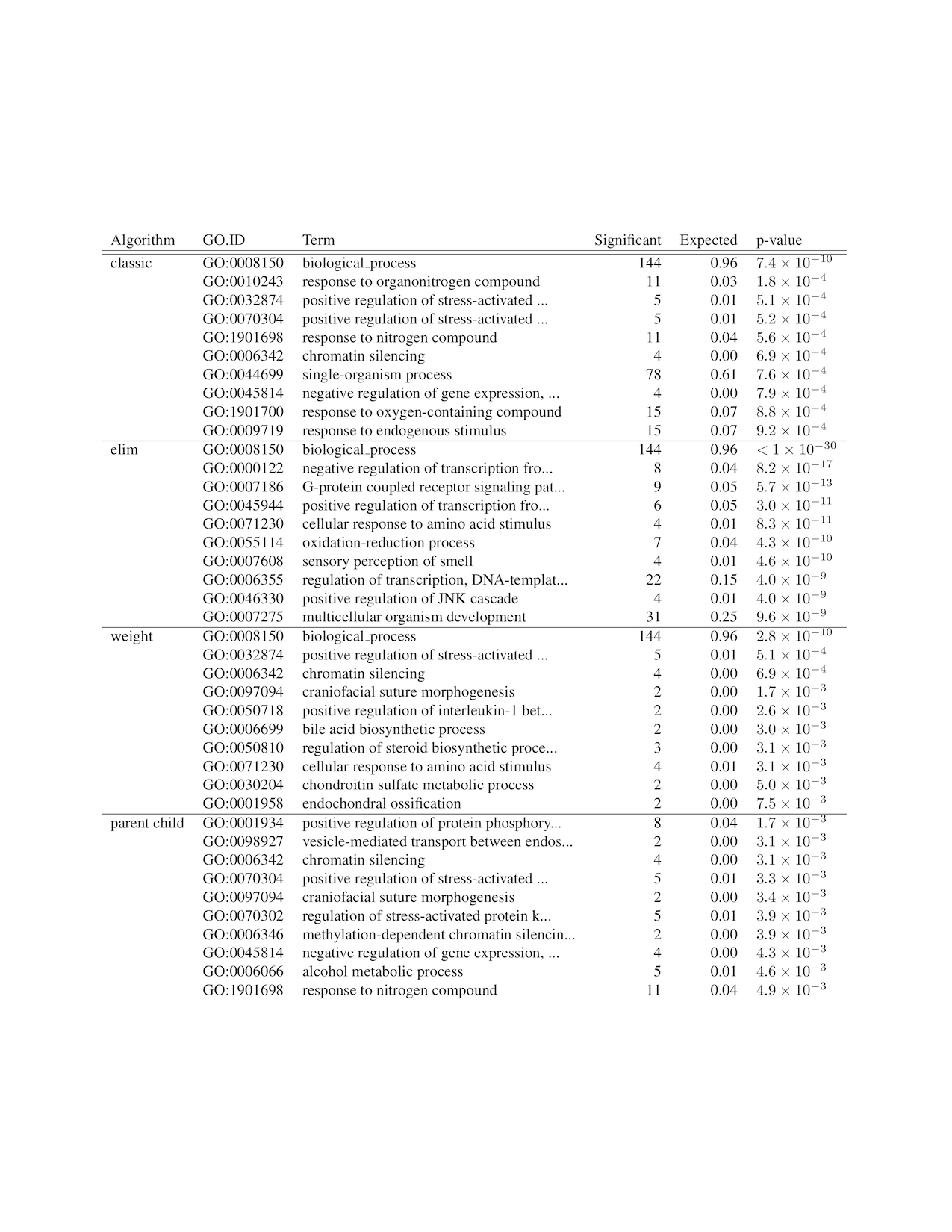

Supplement: S5 Table — P-values for each GO term were calculated using the fisher statistic combined with one of four separate algorithms that each take the GO hierarchy into account (described in Methods). (TIFF) [file pcbi.1006091.s025.tiff]

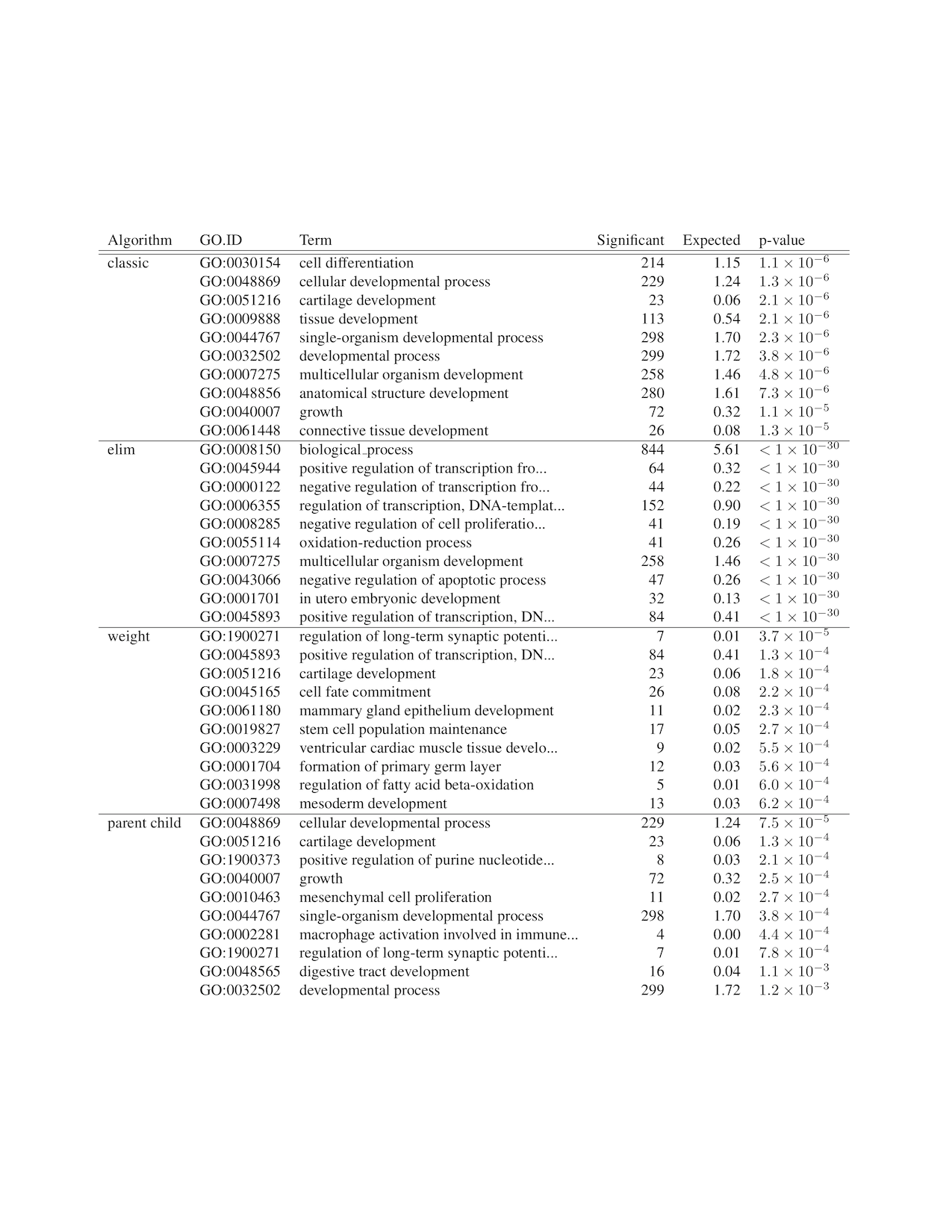

Supplement: S6 Table — P-values for each GO term were calculated using the fisher statistic combined with one of four separate algorithms that each take the GO hierarchy into account (described in Methods). (TIFF) [file pcbi.1006091.s026.tiff]

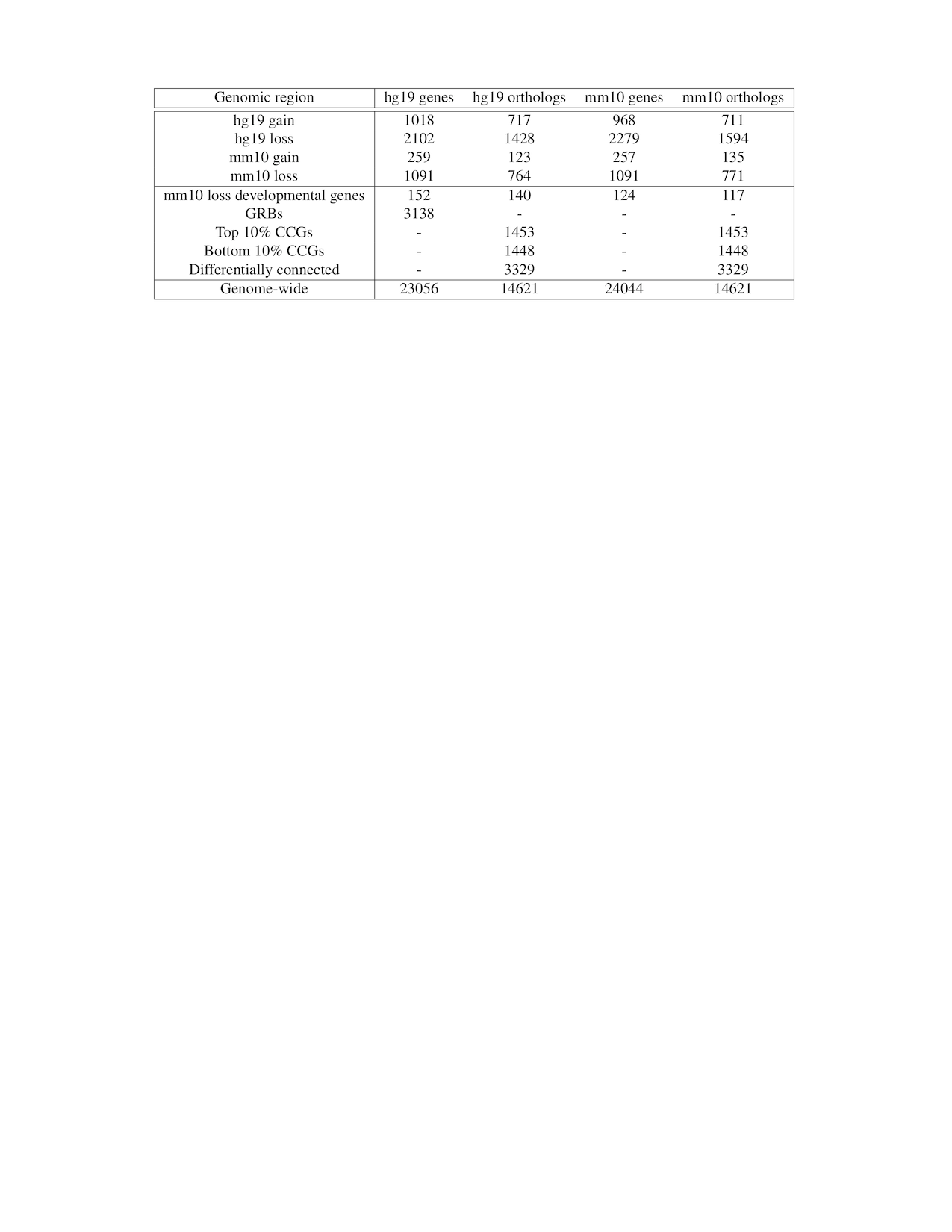

Supplement: S7 Table — mm10 loss developmental genes are genes in mouse loss hotspots that are annotated with developmental process GO terms. GRBs (gene regulatory blocks) are regions in the human genome that are enriched for conserved elements. Top 10% CCGs (commonly co-expressed genes) are the genes with the highest amount of co-expressed orthologs shared between human and mouse, these genes are likely to have conserved expression between both species. Bottom 10% CCGs are the genes with the least amount of co-expressed orthologs shared between human and mouse, these genes are likely to have divergent expression patterns between both species. Differentially connected genes are genes with the highest amount of differential connectivity between human and mouse, these are genes with non-conserved expression patterns. All human and mouse orthologs are one to one. (TIFF) [file pcbi.1006091.s027.tiff]

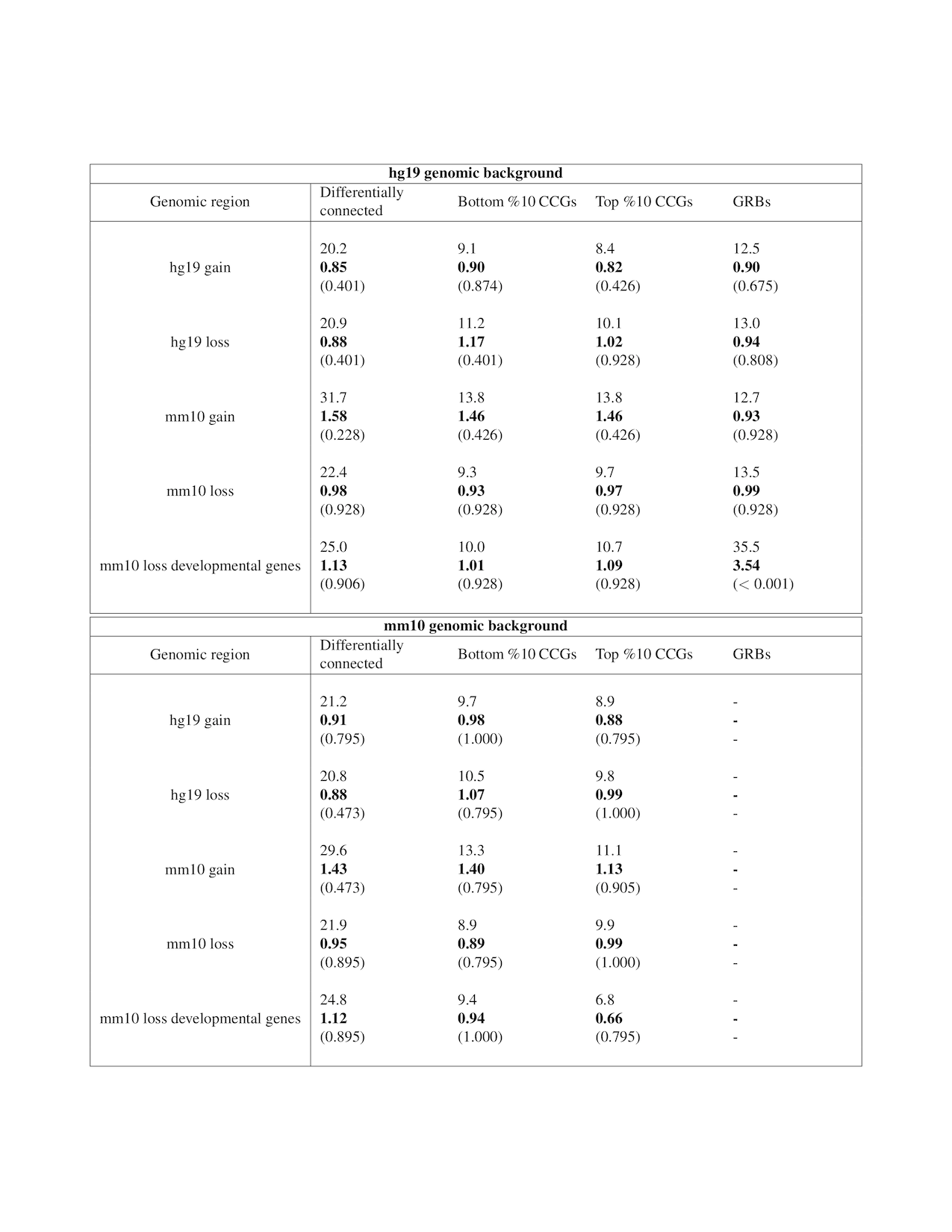

Supplement: S8 Table — For each region comparison the percentage of genes found in gap annotation hotspots was reported. The statistical significance of each overlap was measured using a Fisher’s exact test. Shown in bold font is the odds ratio and shown in brackets is the FDR. (TIFF) [file pcbi.1006091.s028.tiff]
